# Exact Solution of a Constraint Optimization Problem for the Thermoelectric Figure of Merit

**DOI:** 10.3390/ma5030528

**Published:** 2012-03-21

**Authors:** Wolfgang Seifert, Volker Pluschke

**Affiliations:** 1 Institute of Physics, University Halle-Wittenberg, Halle D-06099, Germany; 2 Institute of Mathematics, University Halle-Wittenberg, Halle D-06099, Germany

**Keywords:** thermoelectricity, functionally graded material, figure of merit, device optimization

## Abstract

In the classical theory of thermoelectricity, the performance integrals for a fully self-compatible material depend on the dimensionless figure of merit zT . Usually these integrals are evaluated for constraints z= const. and zT= const., respectively. In this paper we discuss the question from a mathematical point of view whether there is an optimal temperature characteristics of the figure of merit. We solve this isoperimetric variational problem for the best envelope of a family of curves z(T)T .

## 1. Introduction

The compatibility approach [1,2,3] focuses on calculating the relative current density *u* which is defined as the ratio of electric and thermal fluxes, $u=\frac{-j^{2}}{\kappa \nabla T\;\cdot \;j}$ . Note that j and ∇T are vectors. The advantage of using the relative current density u(T) is that the complex thermoelectric (TE) problem can be reduced to a one-dimensional heat flow problem. In particular, this approach can be used as a mathematical basis to analyze the local performance of TE material [4,5].

The total performance (efficiency *η* and coefficient of performance φ , respectively) of a thermogenerator (TEG) or Peltier cooler (TEC) element is obtained by summing up all local contributions in an integral sense as originally proposed by Harman and Honig [6], see also [4,7]:

(1a) $$\begin{array}{c} \text{TEG}\left(T_{\text{s}}\leq T\leq T_{\text{a}}\right):\;\ln(1-\eta )=\int_{T_{\text{a}}}^{T_{\text{s}}}\frac{\eta_{\text{r}}\left(u,T\right)}{T}\;dT=\int_{T_{\text{a}}}^{T_{\text{s}}}\frac{1}{T}\;\frac{u\frac{\alpha }{z}\;\left(1-u\frac{\alpha }{z}\right)}{u\frac{\alpha }{z}+\frac{1}{zT}}\;dT\; \end{array}$$

(1b) $$\begin{array}{c} \text{TEG}\left(T_{\text{a}}\leq T\leq T_{\text{s}}\right):\ln\left(1+\frac{1}{\varphi }\right)=\int_{T_{\text{a}}}^{T_{\text{s}}}\frac{1}{T\varphi_{\text{r}}\left(u,T\right)}\;dT=\int_{T_{\text{a}}}^{T_{\text{s}}}\frac{1}{T}\;\frac{u\frac{\alpha }{z}\;\left(1-u\frac{\alpha }{z}\right)}{u\frac{\alpha }{z}+\frac{1}{zT}}\;dT\;\; \end{array}$$

where we identify one kernel for integrals of both generator and cooler. The model is based on an ideal single element device (prismatic TE element of length *L* and fixed boundary temperatures) without parasitic losses, for more information see [4,5]. Then, the device figure of merit is equal to the traditional material’s figure of merit, $z=\alpha^{2}/\left(\rho \kappa \right)$ , with the Seebeck coefficient (*α*), electrical resistivity (*ρ*), and thermal conductivity (*κ*).

The Integrals (1) can be optimized with respect to the relative current *u*. An optimized *u* represents an optimal ratio between heat flux and electrical current density and hence a maximum performance value given in self-compatible elements by the compatibility factors $u_{\text{opt}}=s^{\left(\text{g}\right)}=\frac{\sqrt{1+zT}-1}{\alpha T}$ of a TEG, but $u_{\text{opt}}=s^{\left(\text{c}\right)}=\frac{-\sqrt{1+zT}-1}{\alpha T}$ of a TEC, firstly introduced by Snyder [1,2]. Thus global maximization is traced back to local optimization [8].

If we assume the ability to achieve full self-compatibility (considering the case of infinite staging) we can apply $u=s^{\left(\text{g}\right)}$ and $u=s^{\left(\text{c}\right)}$ to the Integrals (1), respectively, so that they take their maximal values with the optimal reduced efficiency $\eta_{\text{r,opt}}=\varphi_{\text{r,opt}}=\frac{\sqrt{1+z\;T}-1}{\sqrt{1+z\;T}+1}\;$ for both TEG and TEC [9,10]. Then, fully self-compatible performance parameters $\eta_{\text{sc}}$ and $\varphi_{\text{sc}}$ are given by

(2a) $$\begin{array}{cccccc} \text{TEG} & \;\left(T_{\text{s}}\leq T\leq T_{\text{a}}\right):\;\;\ln\left(1-\eta_{\text{sc}}\right) & = & \int_{T_{\text{a}}}^{T_{\text{s}}}\frac{\eta_{r,opt}}{T}\;dT & =\int_{T_{\text{a}}}^{T_{\text{s}}}\frac{1}{T}\;\frac{\sqrt{1+zT}-1}{\sqrt{1+zT}+1}\;dT & \; \end{array}$$

(2b) $$\begin{array}{cccccc} \text{TEG} & \left(T_{\text{a}}\leq T\leq T_{\text{s}}\right):\ln\left(1+\frac{1}{\varphi_{\text{sc}}}\right) & = & \int_{T_{\text{a}}}^{T_{\text{s}}}\frac{1}{T\varphi_{\text{r,opt}}}\;dT & =\int_{T_{\text{a}}}^{T_{\text{s}}}\frac{1}{T}\;\frac{\sqrt{1+z\;T}+1}{\sqrt{1+z\;T}-1}\;dT & \;\; \end{array}$$

where we identify expressions being monotone with zT in the integrands. For the notation used we refer to [4,5].

We expressly emphasize that the Integrals (2) do not have extremal properties concerning the zT value. Usually they are evaluated analytically for constraints $z=z_{o}=$ const. or $z\;T=k_{o}=$ const., for details see the appendix of [8]. In particular the latter case is easy to handle. We obtain with constant values $\;\eta_{\text{r,opt}}=\varphi_{\text{r,opt}}=\frac{\sqrt{1+k_{o}}-1}{\sqrt{1+k_{o}}+1}$ for the Integrals (2)

(3) $$\eta_{\text{sc}}^{\left(k_{o}\right)}=1-{\left(\frac{T_{\text{s}}}{T_{\text{a}}}\right)}^{\eta_{\text{r,opt}}}\;\;\text{for}\;\text{TEG},\;\;\text{and}\;\;\;\varphi_{\text{sc}}^{\left(k_{o}\right)}={\left[{\left(\frac{T_{\text{s}}}{T_{\text{a}}}\right)}^{1/\varphi_{\text{r,opt}}}-1\right]}^{-1}\;\;\text{for}\;\text{TEC}$$

The question of how to get the best performance can only be answered if we put the constant $k_{o}$ in relation to the TE material characterized by an experimental z(T) . A proof for the relations

(4) $$\eta_{\text{sc}}<\eta_{\text{sc}}^{\left(k_{o}\right)}\;\text{and}\;\varphi_{\text{sc}}<\varphi_{\text{sc}}^{\left(k_{o}\right)}$$

is given in [4], if $k_{o}$ is calculated as the average over temperature of a *monotonically increasing function* z(T)T ,

(5) $$k_{o}=\frac{1}{T_{\text{s}}-T_{\text{a}}}\;\int_{T_{\text{a}}}^{T_{\text{s}}}z\left(T\right)\;T\;dT$$

Then we get

(6) $$\begin{array}{cccc} \text{TEG}: & \;\;1-\exp\left(-\int_{T_{\text{s}}}^{T_{\text{a}}}\frac{1}{T}\;\frac{\sqrt{1+z(T)\;T}-1}{\sqrt{1+z(T)\;T}+1}\;dT\right) & \leq & \;1-{\left(\frac{T_{\text{s}}}{T_{\text{a}}}\right)}^{\frac{\sqrt{1+k_{o}}-1}{\sqrt{1+k_{o}}+1}} \end{array}$$

(7) $$\begin{array}{cccc} \text{TEG}: & \;\;\;{\left[\exp\left(\int_{T_{\text{a}}}^{T_{\text{s}}}\frac{1}{T}\frac{\sqrt{1+z(T)\;T}+1}{\sqrt{1+z(T)\;T}-1}\;dT\right)-1\right]}^{-1} & \leq & \;{\left[{\left(\frac{T_{\text{s}}}{T_{\text{a}}}\right)}^{\frac{\sqrt{1+k_{o}}+1}{\sqrt{1+k_{o}}-1}}-1\right]}^{-1} \end{array}$$

Equality holds if z(T)T= const. If z(T)T is decreasing, however, the above inequalities in general do not hold. Hence, we look for an optimal z(T)T where $\eta_{\text{sc}}>\eta_{\text{sc}}^{\left(k_{o}\right)}$ and $\varphi_{\text{sc}}>\varphi_{\text{sc}}^{\left(k_{o}\right)}$ , respectively, and $\eta_{\text{sc}}$ , $\varphi_{\text{sc}}$ will be maximal. Since the integrals cannot be optimized for arbitrary zT we consider a constraint optimization problem including Condition (5). The solution enlightens the role of the constraint zT= const. which is often used in practice.

## 2. Linear Functions *k(T) = z(T)T*

Before turning to the general problem, let us examine linear functions k(T)=z(T)T .

We define straight lines k(T) by the formula

(8) $$\begin{array}{c} k\left(T\right)=\frac{2\;k_{o}}{1+\xi }\left[\xi +\left(1-\xi \right)\;\frac{T-T_{s}}{T_{a}-T_{s}}\right]\;\text{with}\;\xi =\frac{k_{\text{s}}}{k_{\text{a}}} \end{array}$$

and boundary values

$$k_{\text{s}}=\xi \;\frac{2\;k_{o}}{1+\xi }\;,\;\;k_{\text{a}}=\frac{2\;k_{o}}{1+\xi }$$

**Figure 1:**
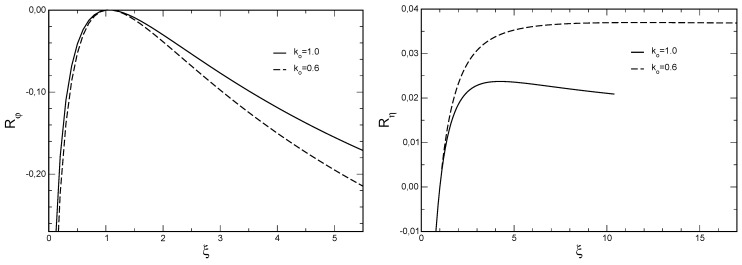
Relative performance increase *R* as function of the parameter *ξ*: left: $R_{\eta }$ $=\eta_{sc}/\eta_{sc}^{\left(k_{o}\right)}-1$ for TEG ( $T_{a}=600\;K,\;T_{s}=300\;K$ ) for $k_{o}=1$ (solid curve, optimal efficiency $\eta_{sc,opt}$ at $\xi_{opt}=4.2$ ) and $k_{o}=0.6$ (dashed curve, $\eta_{sc,opt}$ at $\xi_{opt}=11.9$ , curve slowly decreasing for $\xi >\xi_{opt}$ as long as $k_{o}>0.5$ ); right: $R_{\varphi }=\varphi_{sc}/\varphi_{sc}^{\left(k_{o}\right)}-1$ for TEC ( $T_{a}=270\;K,\;T_{s}=300\;K$ ) for $k_{o}=1$ (solid) and $k_{o}=0.6$ (dashed), optimal coefficient of performance $\varphi_{sc,opt}=1.0002$ $\varphi_{sc}^{\left(k_{o}\right)}$ at $\xi_{opt}=1.055$ for both curves.

**Figure 2:**
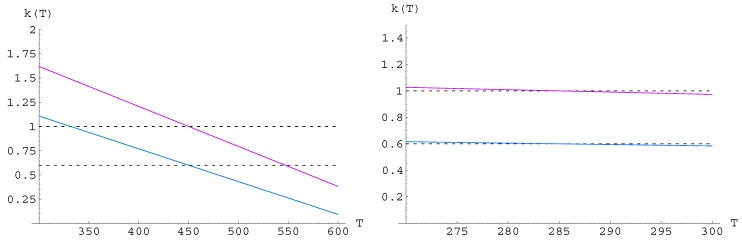
Optimal straight line $k_{opt}\left(T\right)=k\left(T,\xi_{opt}\right)$ plotted with the optimal parameter $\xi_{opt}$ derived from Figure 1: left (TEG): $\xi_{opt}=4.2$ for $k_{o}=1$ (purple) and $\xi_{opt}=11.9$ for $k_{o}=0.6$ (blue); right (TEC): $\xi_{opt}\approx 1$ (from $\xi_{opt}=1.054$ for $k_{o}=0.1$ to $\xi_{opt}=1.062$ for $k_{o}=10$ , with $\xi_{opt}=1.055$ for $k_{o}=0.6$ and $k_{o}=1$ ).

The goal is to estimate the optimal $\xi_{opt}$ which gives maximum performances $\eta_{\text{sc}}$ and $\varphi_{\text{sc}}$ , respectively. Exemplarily, Figure 1 shows the results for $k_{o}=0.6$ and $k_{o}=1$ for both TEG and TEC. Having found $\xi_{opt}$ , the optimal function $k_{opt}\left(T\right)=k\left(T,\xi_{opt}\right)$ can be derived, see Figure 2. Note that $k_{opt}\left(T\right)$ is decreasing with temperature for TEG (leading to a small performance increase of about 4% for $k_{o}=0.6$ ), but the maximal coefficient of performance of a TEC is very close to k=zT= const. when considering straight lines k(T) .

More generally, one can prove for straight lines: For both a TEG and TEC, the performance increases if we cross the function k=zT= const. from increasing straight lines to decreasing straight lines. For TEG the existence of a maximal performance value in the class of straight lines depends on $k_{o}$ and on the quotient $T_{a}/T_{s}$ . There is a maximum in efficiency if $k_{o}$ is large enough and $T_{a}/T_{s}$ is not too large. Otherwise, the performance $\eta_{sc}$ increases the stronger k(T)=z(T)T is falling. We see this effect in our example, see left subfigure of Figure 1: For $k_{o}=1$ (solid curve) a clear maximum of *η* appears at $\xi_{opt}=4.2$ . For a smaller $k_{o}=0.6$ the maximum is not so manifest (dashed curve). This $k_{o}$ is only a little bit larger than the critical value $k_{o}=0.5$ for $T_{a}/T_{s}=2$ , where a maximal performance value no longer exists. For $k_{o}<0.5$ the dashed curve in Figure 1, left side, would be monotonically increasing for all ξ>0 .

**Figure 3:**
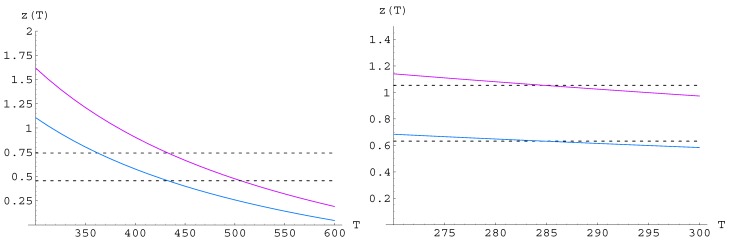
Optimal figure of merit $z_{opt}\left(T\right)$ ; left TEG, right TEC (for boundary temperatures and colours see the legends of Figure 1 and Figure 2).

For a TEC we have a different situation. There is always a maximal coefficient of performance $\varphi_{sc,opt}$ in the class of straight lines k(T) for some $\xi_{opt}>1$ (decreasing *k*) independent of $k_{o}$ and $T_{s}/T_{a}$ . In general, however, this optimal value $\xi_{opt}>1$ is very close to ξ=1 and in our Figure 1 (right subfigure) it seems that this might be 1. Actually, the maximal value of $\varphi_{sc,opt}$ is attained at $\xi_{opt}=1.055$ and exceeds $\varphi_{sc}^{\left(k_{o}\right)}$ by only 0.02% . From these results, the optimal figure of merit $z_{opt}\left(T\right)=k\left(T,\xi_{opt}\right)/T$ can be calculated, see Figure 2 and Figure 3. The large effect for TEG (left) is obviously due to the fact that the temperature range of ΔT=300K for TEG is ten times larger than for TEC.

In the next section we derive a condition for the optimal profile k(T)=z(T)T . It turns out that this optimal function is not a straight line, but the situation is similar to the case of straight lines described above. The optimal function is decreasing again, and there is the same qualitative connection between $k_{o}$ and the existence of an optimal profile. Especially for a TEC, the restriction to straight lines will be a good approximation of the solution.

## 3. Isoperimetric Variational Problem

In this section we solve the two isoperimetric variational problems

(9a) $$\begin{array}{ccc} \text{TEG} & \;(T_{\text{s}}\leq T\leq T_{\text{a}}):\; & \int_{T_{\text{s}}}^{T_{\text{a}}}\frac{1}{T}\;\frac{\sqrt{1+zT}-1}{\sqrt{1+zT}+1}\;dT\;⟶\;\text{Max}\; \end{array}$$

(9b) $$\begin{array}{ccc} \text{TEG} & \;(T_{\text{a}}\leq T\leq T_{\text{s}}):\; & \int_{T_{\text{a}}}^{T_{\text{s}}}\frac{1}{T}\;\frac{\sqrt{1+z\;T}+1}{\sqrt{1+z\;T}-1}\;dT\;⟶\;\text{Min}\; \end{array}$$

with Constraint (5). The corresponding Lagrange functions (with Euler multiplicator *λ*) are

(10a) $$\begin{array}{cc} L(T,z,\lambda ) & =\frac{1}{T}\;\frac{\sqrt{1+zT}-1}{\sqrt{1+zT}+1}+\frac{\lambda }{T_{2}-T_{1}}\;zT \end{array}$$

and

(10b) $$\begin{array}{cc} L(T,z,\lambda ) & =\frac{1}{T}\;\frac{\sqrt{1+zT}+1}{\sqrt{1+zT}-1}+\frac{\lambda }{T_{2}-T_{1}}\;zT\;, \end{array}$$

respectively, where $T_{1}:\;=\min\left\{T_{\text{s}},T_{\text{a}}\right\}$ and $T_{2}:\;=\max\left\{T_{\text{s}},T_{\text{a}}\right\}$ . Hence, Euler’s equation reduces to ∂L/∂z=0 together with Condition (5). Differentiating Equation (10a) and Equation (10b) we obtain the following necessary relation for the optimal profile k(T)=z(T)T to Problem (9),(5).

**Theorem 1.**  *Let $z=z_{\max}$ or $z=z_{\min}$ be an optimal function that maximizes the Integral (9a) or minimizes the Integral (9b), respectively, under Restriction (5). Then it fulfills the Equations*

(11a) $$\begin{array}{cccc} TEG: & \; & T\;\sqrt{1+z_{\max}\left(T\right)T}{\left(\sqrt{1+z_{\max}\left(T\right)T}+1\right)}^{2} & =\mu \; \end{array}$$

(11b) $$\begin{array}{cccc} TEC: & \; & T\;\sqrt{1+z_{\min}\left(T\right)T}{\left(\sqrt{1+z_{\min}\left(T\right)T}-1\right)}^{2} & =\mu \; \end{array}$$

*where $\mu =\mu (k_{o})$ is a real constant depending on $k_{o}$ by means of*

(12) $$\frac{1}{T_{2}-T_{1}}\;\int_{T_{1}}^{T_{2}}z_{\max/\min}\left(T\right)\;T\;dT=k_{o}\;$$

In order to calculate the optimal solution $z_{\max/\min}\left(T\right)$ we have to solve the System (11), (12). Substituting $x:\;=\sqrt{1+z(T)T}$ , Equations (11) simplify to

(13) $$x{\left(x+1\right)}^{2}=\mu /T\;\text{and}\;x{\left(x-1\right)}^{2}=\mu /T$$

**Figure 4:**
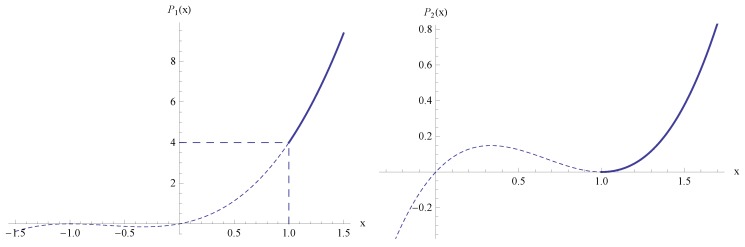
Graph of polynomials $P_{1}\left(x\right)=x{\left(x+1\right)}^{2}$ and $P_{2}\left(x\right)=x{\left(x-1\right)}^{2}$ , see Equation (13).

Since z(T)T>0 we look for solutions x>1 of Equations (13). From the graph of the polynomials $P_{1}\left(x\right)=x{\left(x+1\right)}^{2}$ and $P_{2}\left(x\right)=x{\left(x-1\right)}^{2}$ (see Figure 4) we find that for fixed μ>0 the first Equation of (13) has exactly one real solution $x_{\mu }\left(T\right)>1$ if $P_{1}\left(x\right)>4$ . This implies the restriction μ/T>4 . The second Equation of (13) has exactly one real solution $x_{\mu }\left(T\right)>1$ for all μ,T>0 .

Then, resubstituting *x* by $z\left(T\right)T=x^{2}-1$ for fixed *μ* with μ/T>4 , we obtain a unique positive solution of Equation (11a)

(14) $$\begin{array}{c} k_{\mu }\left(T\right)=z_{\mu }\left(T\right)T=-1+\frac{1}{9}\left(-2+\frac{2^{1/3}}{{\left(2+\frac{27\mu }{T}+3\sqrt{3}\;\sqrt{\frac{4\mu }{T}+\frac{27\mu^{2}}{T^{2}}}\right)}^{1/3}}\right.\\ +{\left.\frac{{\left(2+\frac{27\mu }{T}+3\sqrt{3}\;\sqrt{\frac{4\mu }{T}+\frac{27\mu^{2}}{T^{2}}}\right)}^{1/3}}{2^{1/3}}\right)}^{\;\;2}\; \end{array}$$

An analogue formula holds for the unique nonnegative solution of Equation (11b). To calculate the Representation (14) an algebra tool (e.g., Mathematica) can be helpful.

It remains to determine the constant *μ*. We have to choose it in a way that $k_{\mu }\left(T\right)=z_{\mu }\left(T\right)T$ from Equation (14) fulfills Condition (12). The question whether we can find such a *μ* is answered by the following theorem:

**Theorem 2.** 

*(i)*  In case of a TEG there is a constant ${\bar{k}}_{o}$ such that the following holds: If $k_{o}\geq {\bar{k}}_{o}$ there exists a unique $\mu =\mu^{*}$ such that the function $k_{\mu^{*}}$ defined by Equation (14) fulfills Equation (11a) as well as Condition (12). Hence, $z_{\max}\left(T\right):=z_{\mu^{*}}\left(T\right)$ . The corresponding $k_{\max}\left(T\right)=z_{\max}\left(T\right)T$ is nonnegative on the interval $\left[T_{1},T_{2}\right]$ , strictly monotonically decreasing and convex. If $0<k_{o}<{\bar{k}}_{o}$ there is no constant μ such that the corresponding solution $z_{\mu }\left(T\right)$ of Equation (11a) is nonnegative for every $T\in [T_{1},T_{2}]$ and fulfills Equation (12). In this case there is no optimal profile.
*(ii)*  In case of a TEC for every $k_{o}>0$ there exist a unique $\mu =\mu^{*}$ and a unique function $z_{\min}\left(T\right):=z_{\mu^{*}}\left(T\right)$ which solve Equations (11b) and (12). The corresponding $k_{\min}\left(T\right)=z_{\min}\left(T\right)T$ is nonnegative, strictly monotonically decreasing and convex.

*Proof.* 

(i) Let $k_{\mu }$ be the (unique) solution of Equation (11a) for fixed μ>0 given by Equation (14). We rewrite Equation (11a) by
(15) $$\sqrt{1+k_{\mu }\left(T\right)}{\left(\sqrt{1+k_{\mu }\left(T\right)}+1\right)}^{2}=\frac{\mu }{T}$$
  and observe that the right hand side is strictly monotonically decreasing w.r.t. *T* for every fixed μ>0 . Hence, $k_{\mu }$ is a strictly decreasing function as well. This yields the nonnegativity of $k_{\mu }\left(T\right)$ if $k_{\mu }\left(T_{2}\right)\geq 0$ which is fulfilled if
$$\frac{\mu }{T_{2}}=\sqrt{1+k_{\mu }\left(T_{2}\right)}{\left(\sqrt{1+k_{\mu }\left(T_{2}\right)}+1\right)}^{2}\geq 4.$$
  Therefore, we have the condition $\mu \geq \bar{\mu }:=4T_{2}$ for the nonnegativity of $k_{\mu }\left(T\right)$ for all $T\in [T_{1},T_{2}]$ . We define now
$$av\left(\mu \right):=\frac{1}{T_{2}-T_{1}}\;\int_{T_{1}}^{T_{2}}k_{\mu }\left(T\right)\;dT$$
  and ${\bar{k}}_{o}:=av\left(\bar{\mu }\right)$ . By the same argument as above we obtain from Equation (15) $k_{\mu_{1}}\left(T\right)<k_{\mu_{2}}\left(T\right)$ if $\bar{\mu }\leq \mu_{1}<\mu_{2}$ for every fixed *T*. Consequently, $av\left(\mu_{1}\right)<av\left(\mu_{2}\right)$ if $\bar{\mu }\leq \mu_{1}<\mu_{2}$ , *i.e.*, $av:\left[\bar{\mu },\infty \right)\to R_{+}$ is strictly monotonically increasing. Moreover, av is a continuous function of *μ*. This implies for every $k_{o}\geq {\bar{k}}_{o}$ the existence of a unique value $\mu =\mu^{*}\geq \bar{\mu }$ such that $av\left(\mu^{*}\right)=k_{o}$ , hence Equation (12). For $k_{o}<{\bar{k}}_{o}$ there is no $\mu \geq \bar{\mu }$ such that $av\left(\mu \right)=k_{o}$ . Therefore there is no nonnegative function $k_{\mu }\left(T\right)=z_{\mu }\left(T\right)T$ which fulfills Equation (11a) and (12), which means that there is no extremal solution for the variational Problem (9a) with Constraint (5).
(ii) By the discussion above it is obvious that in the case of a TEC there is a unique and nonnegative solution $k_{\mu }\left(T\right)=z_{\mu }\left(T\right)T$ of Equation (11b) for every fixed μ>0 . The representation
$$\sqrt{1+k_{\mu }\left(T\right)}{\left(\sqrt{1+k_{\mu }\left(T\right)}-1\right)}^{2}=\frac{\mu }{T}$$
  of Equation (11b) yields that $k_{\mu }$ is strictly monotonically decreasing with respect to *T* and, moreover, that $k_{\mu }\left(T\right)$ increases for fixed *T* if *μ* increases. This implies the strict monotonicity of $av:\left(0,\infty \right)\to R_{+}$ . Furthermore, as illustrated in Figure 4, if *μ* decreases to zero then $k_{\mu }$ decreases to zero (since x↘1 ), hence av(μ)↘0 . Consequently, for every $k_{o}>0$ there is a unique $\mu =\mu^{*}$ such that the solution $z_{\min}\left(T\right)T:=k_{\mu^{*}}\left(T\right)$ of Equation (11b) fulfills the condition $av\left(\mu^{*}\right)=k_{o}$ , *i.e.*, it is the optimal solution of Equations (9b) and (5).

The proof of convexity of the optimal functions $k_{\mu^{*}}$ is given in the appendix. ☐

**Remark 1.** 

*1*. *The observations in Section 2 on linear functions reflect the general result. Certain monotonically decreasing straight lines yield a better performance than the increasing ones. Moreover, as discussed in Section 2, also in the case of linear functions k(T) there is a critical value ${\bar{k}}_{o}>0$ of $k_{o}$ for TEG, where we have no optimal linear function below of it. For a TEC such a critical ${\bar{k}}_{o}$ does not occur. There we have an optimal performance in the class of linear function for every $k_{o}>0$ .*
*2*. *It is obvious that also $z_{opt}$ will be strictly monotonically decreasing since $k_{opt}\left(T\right)=z_{opt}\left(T\right)T$ has this property. Even more, $z_{opt}$ will be a convex function. This can be justified by the following calculation using strict convexity of $k_{opt}\left(T\right)=z_{opt}\left(T\right)T$ :*
$$0<k_{opt}^{\prime \prime }\left(T\right)={\left(z_{opt}\left(T\right)T\right)}^{\prime \prime }={\left(z_{opt}^{\prime }\left(T\right)T+z_{opt}\left(T\right)\right)}^{\prime }=2z_{opt}^{\prime }\left(T\right)+z_{opt}^{\prime \prime }\left(T\right)T$$
  *Since $z_{opt}^{\prime }\left(T\right)<0$ for all T this can only be fulfilled if $z_{opt}^{\prime \prime }\left(T\right)>0$ which means convexity.*

In order to calculate the optimal TEG or TEC profile for given $k_{o}$ , we now have to determine the constant *μ* such that the solution $k_{\mu }\left(T\right)=z_{\mu }\left(T\right)T$ of Equation (11) satisfies Condition (12). Since we cannot evaluate the integral of a function like Equation (14) explicitly, we have to use numerical methods to solve the equation $av\left(\mu \right)=k_{o}$ for *μ*. Due to the strict monotonicity of av(μ) , a standard numerical solver will work.

Now we compare the best linear functions from Section 2 with the optimal profile corresponding to Theorem 2. Again we choose $k_{o}=1$ and $k_{o}=0.6$ for a TEG and a TEC, respectively. We start with a TEG with $T_{s}=300K$ and $T_{a}=600K$ like in Section 2.

We compare the corresponding values of the efficiency $\eta_{\text{sc}}$ for the three cases that $k\left(T\right)=k_{o}$ is a constant, $k\left(T\right)=k\left(T,\xi_{opt}\right)$ is the best linear function of Section 2 and $k\left(T\right)=k_{\max}\left(T\right)=z_{\max}\left(T\right)T$ is the global maximum of the variational Problem (9a),(5), see Table 1:

**Table 1:** Self-compatible efficiency of a TEG with $T_{s}=300K$ and $T_{a}=600K$ .

TEG	$k_{o}=1$		$k_{o}=0.6$	
	$\eta_{\text{sc}}$	$\eta_{\text{sc}}/\eta_{\text{sc}}^{\left(k_{o}\right)}$	$\eta_{\text{sc}}$	$\eta_{\text{sc}}/\eta_{\text{sc}}^{\left(k_{o}\right)}$
constant function $k\left(T\right)=k_{o}$	0.112126	1.00000	0.077873	1.00000
linear function $k\left(T\right)=k\left(T,\xi_{opt}\right)$	0.114786	1.02372	0.080752	1.03697
optimal function $k\left(T\right)=k_{\max}\left(T\right)$	0.114855	1.02434	0.080829	1.03796

**Figure 5:**
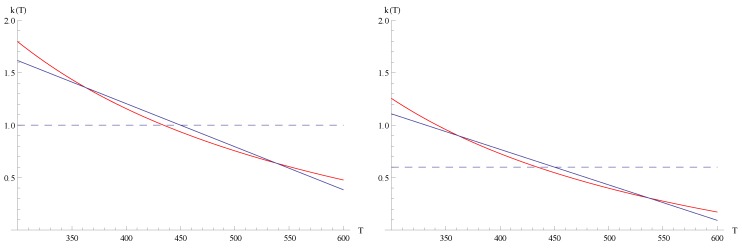
Optimal functions $k_{\max}\left(T\right)$ (red) compared with the best straight line $k(T,\xi_{opt})$ (blue) from Figure 2 plotted with the optimal parameter $\xi_{opt}$ derived from Figure 1. left: $\xi_{opt}=4.2$ for $k_{o}=1$ ; right: $\xi_{opt}=11.9$ for $k_{o}=0.6$ .

**Figure 6:**
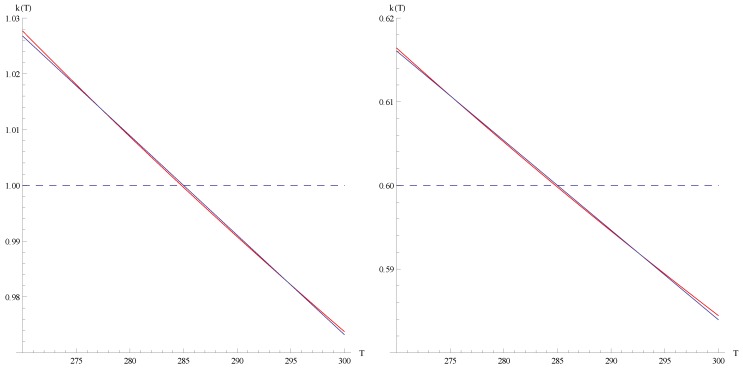
Optimal monotonic functions $k_{\min}\left(T\right)$ (red) compared with the best straight line $k(T,\xi_{opt})$ (blue) from Figure 2 plotted with the optimal parameter $\xi_{opt}=1.055$ derived from Figure 1. left: $k_{o}=1$ ; right: $k_{o}=0.6$ . Please note the scaling of the y-axis.

Both from the above table and Figure 5 we see that the best straight line is a good approximation for the optimal profile. The optimal function $k_{\max}\left(T\right)=z_{\max}\left(T\right)T$ , due to Theorem 2, yields only a minimal increase in performance compared with the best linear function. This effect becomes even more apparent in the case of TEC which will be considered now (see Figure 6). Like in Section 2 we choose again $T_{a}=270\;K$ and $T_{s}=300\;K$ .

We observe that there is almost no difference between the best linear function and the optimal profile $k_{\min}\left(T\right)$ which can be distinguished only thanks to the different scaling of the axes. Moreover, the scaling should not hide the fact that both functions nearly coincide with the constant $k\left(T\right)=k_{0}$ . Again we compare the maximal values of the coefficient of performance $\varphi_{\text{sc}}$ for the three cases that $k\left(T\right)=k_{o}$ is a constant, $k\left(T\right)=k\left(T,\xi_{opt}\right)$ is the best linear function of Section 2 and $k\left(T\right)=k_{\min}\left(T\right)=z_{\min}\left(T\right)T$ is the global minimum of the variational Problem (9b),(5), respectively (Table 2):

**Table 2:** Self-compatible coeff. of performance of a TEC with $T_{a}=270K$ and $T_{s}=300K$ .

TEC	$k_{o}=1$		$k_{o}=0.6$	
	$\varphi_{\text{sc}}$	$\varphi_{\text{sc}}/\varphi_{\text{sc}}^{\left(k_{o}\right)}$	$\varphi_{\text{sc}}$	$\varphi_{\text{sc}}/\varphi_{\text{sc}}^{\left(k_{o}\right)}$
constant function $k\left(T\right)=k_{o}$	1.17929125	1.0000000	0.68419337	1.0000000
linear function $k\left(T\right)=k\left(T,\xi_{opt}\right)$	1.17955485	1.0002235	0.68438545	1.0002803
optimal function $k\left(T\right)=k_{\min}\left(T\right)$	1.17955497	1.0002236	0.68438554	1.0002804

Here we see that for a TEC the constant function $k\left(T\right)=z\left(T\right)T=k_{o}$ is a good choice, since there is only an insignificant increase of $\varphi_{\text{sc}}$ for the optimal function $k_{\min}\left(T\right)$ .

## 4. Discussion and Conclusions

The material’s figure of merit *z* gathers as a primary parameter the different transport coefficients of thermoelectrics, leading to an efficient classification of the various TE materials. The dimensionless zT in turn appears in a variety of thermodynamic expressions [11]. At a first glance the presence of the temperature in the expression of the dimensionless figure of merit may be strange since *T* is not a material property, but an intensive parameter which partly defines the working conditions. Nevertheless, one should notice that, in terms of thermodynamic optimization, the material properties are nothing without considering the available exergy of the working system, for more information see [5,11]. The figure of merit is clearly the central term for TE material engineering.

A general rule is that if a material is good (high zT ) then it is good in both TEG and cooler applications. However, the question is whether the constraint zT= const. can be considered as a local condition for an optimal material. The counter argument usually advanced is that the Seebeck coefficient α(T) and the electric conductivity σ(T) have opposite shapes, which has given rise to the hope that a down-opened parabola z(T) (resp. z(x) ) could be close to the optimal condition. This hope is not fulfilled when considering the problem from a mathematical point of view. In the performance integrals, z(T)T is representing an internal degree of freedom that must be fixed by an upper limit or similar constraint in order to prevent that global performance diverges. Doing so, a constraint optimization problem for the thermoelectric figure of merit has been formulated and solved. As the result we obtain convex, optimal functions k(T)=z(T)T , slightly falling with temperature, for both TEG and TEC. It is well-known that curves k(T)=z(T)T falling with temperature are practically not usable for most materials. However, it has turned out that the optimal function k(T) is almost a constant $k\left(T\right)=k_{o}$ for a TEC and close to this constant function for a TEG, respectively (see Table 1 and Table 2). This fact underlines the importance of the constraint $zT=k_{o}=$ const. which is often used in practice; usually this constraint can only be reached approximately.

## Appendix

We complete the proof of Theorem 2 with the following lemma:

**Lemma A1.** 
*Every solution $k_{\mu }\left(T\right)=z\left(T\right)T$ of Equation (11a) (TEG) or Equation (11b) (TEC), respectively, is a convex function.*

*Proof.* We simultaneously deal with both Equation (11) and differentiate

(11)
$$T\sqrt{1+k_{\mu }\left(T\right)}{\left(\sqrt{1+k_{\mu }\left(T\right)}\pm 1\right)}^{2}=\mu$$

with respect to *T* for fixed μ>0 and obtain

$$\sqrt{1+k_{\mu }}{\left(\sqrt{1+k_{\mu }}\pm 1\right)}^{2}+\frac{T{\left(\sqrt{1+k_{\mu }}\pm 1\right)}^{2}}{2\sqrt{1+k_{\mu }}}\;k_{\mu }^{\prime }\left(T\right)+T\left(\sqrt{1+k_{\mu }}\pm 1\right)\;k_{\mu }^{\prime }\left(T\right)=0$$

Now we expand all items in a way that the left hand side of Equation (11) appears in the numerator of every fraction and replace it by *μ*,

$$\frac{\mu }{T}+\left(\frac{\mu }{2(1+k_{\mu })}+\frac{\mu }{\sqrt{1+k_{\mu }}\left(\sqrt{1+k_{\mu }}\pm 1\right)}\right)k_{\mu }^{\prime }\left(T\right)=0$$

or, equivalently,

$$k_{\mu }^{\prime }\left(T\right)\left[\frac{1}{2(1+k_{\mu }\left(T\right))}+\frac{1}{\sqrt{1+k_{\mu }\left(T\right)}\left(\sqrt{1+k_{\mu }\left(T\right)}\pm 1\right)}\right]=-\;\frac{1}{T}\;$$

Since the item […] in the brackets is positive we have $k_{\mu }^{\prime }\left(T\right)<0$ for all $T\in [T_{1},T_{2}]$ , hence we see again that our optimal solution is monotonically decreasing. Moreover, we have

$$k_{\mu }^{\prime }\left(T\right)=-{\left[\;\underset{a(T)}{\underset{︸}{\frac{T}{2(1+k_{\mu }\left(T\right))}}}+\underset{b(T)}{\underset{︸}{\frac{T}{\sqrt{1+k_{\mu }\left(T\right)}\left(\sqrt{1+k_{\mu }\left(T\right)}\pm 1\right)}}}\;\right]}^{-1}$$

The items a(T) and b(T) are strictly increasing since $k_{\mu }\left(T\right)$ is decreasing w.r.t. *T*. This implies that ${\left[a\left(T\right)+b\left(T\right)\right]}^{-1}$ is strictly decreasing and $k_{\mu }^{\prime }\left(T\right)=-{\left[a\left(T\right)+b\left(T\right)\right]}^{-1}$ is strictly increasing again. This means strict convexity of $k_{\mu }$ . ☐
